# Identification and expression profiling of the bone morphogenetic protein gene family based on pearl culture in mantle and visceral mass of *Hyriopsis cumingii*

**DOI:** 10.3389/fvets.2024.1445594

**Published:** 2024-08-21

**Authors:** Yiwen Chen, Shijun Liu, Yuanbin Yao, Junlin Sun, Xiaofeng Chen, Xiangyang Yu, Xingrong Xuan, Xiangli Bian, Wenjuan Li

**Affiliations:** ^1^Key Laboratory of Freshwater Aquatic Genetic Resources, Ministry of Agriculture and Rural Affairs, Shanghai Ocean University, Shanghai, China; ^2^Science and Technology Service Center, Ministry of Science and Technology, Shanghai Mugao Biotechnology Co., Ltd., Shanghai, China; ^3^Shanghai Engineering Research Center of Aquaculture, Shanghai Ocean University, Shanghai, China; ^4^Department of Pediatric, Shanghai Sixth People's Hospital, Shanghai, China

**Keywords:** *Hyriopsis cumingii*, bone morphogenetic proteins, identification, expression analysis, pearl culture

## Abstract

Bone morphogenetic proteins (BMPs) play an important biological role in pearl biomineralization in pearl mussels. In this study, based on the genome data of the triangular sail mussel (*Hyriopsis cumingii*), the genome-wide identification and bioinformatic analysis of BMP gene family were performed, and the expression pattern of the BMP genes was investigated by the insertion experiments. The results showed that a total of 12 BMP gene family members (*BMP2a/2b*, *BMP3*, *BMP5a/5b, BMP7a/7b/7c*, *BMP9*, *BMP10a/10b*, and *BMP11*) were identified, which were unevenly distributed on chromosome 3/14/18, encoding 169–583 amino acids, with molecular weights ranging from 19.32 to 65.99 kDa. *BMP2a*, *BMP7b*, and *BMP10a* were distributed, respectively, in the cytoplasm, endoplasmic reticulum and mitochondria, others were distributed in the nucleus. qRT-PCR results showed the significant tissue specificity in BMPs gene expression. The HcBMPs were differentially expressed in the mantle and visceral mass, and the expression level was higher in the visceral mass. The expressing trends of HcBMPs were not consistent between the mantle and visceral mass insertion, suggesting that HcBMPs may perform different functions. We also found that insertion surgery in the mantle and visceral mass significantly alters the expression profiling of the BMP gene family. Insertion of the mantle induced the biomineralization function of *BMP2a*, *BMP7a*, and *BMP7b*, while *BMP3* and *BMP10b* played opposite roles in visceral mass insertion. Visceral mass insertion could suppress *BMP9* expression at 5 d and *BMP5b* expression at 90 d after insertion This work lays the foundation and data support for the preliminary elucidation of regulatory role and mechanism of HcBMPs in the pearl-cultivating process of mantle and visceral mass.

## 1 Introduction

The shell of the mussel serves as its exoskeleton, which can protect the soft internal tissues and visceral organs (1), and is a product of its biomineralization (2). In addition, pearls are formed by the secretion of an organic matrix from the mantle of pearl mussel, which forms calcium carbonate and undergoes biomineralization. They are identical in composition to that of the shell, but are structurally different (3), and the formation process is regulated at the genetic level. The triangular sail mussel (*Hyriopsis cumingii*) is the most important freshwater pearl-breeding mussel in China (4), and the pearls cultured are one of the typical representatives of biomineralization products. When the mussel is stimulated by artificially inserted nucleus and pieces of donor mantle, the epithelial cells on the outer side of the mantle at the insertion point are stimulated by the foreign substances. They will interact with the mantle pieces, proliferate continuously and gradually enclose the inserted nucleus, forming a sac-like structure called the pearl sac, which continuously secretes organic macromolecules and ions deposited on the surface of the nucleus (5). At present, the common nucleus insertion parts of *H. cumingii* are mantle and visceral mass, the mantle is relatively thin, so the nucleus is easy to penetrate and fall off, and the rate of pearl formation is low (6). The visceral mass, as a potential site for cultivating round and large pearls, is characterized by its large size and multiple insertion sites, which can form high-quality pearls with good luster, large particles and round shapes (7), but there have been fewer studies on the regulatory mechanisms of *H. cumingii* after the insertion of nucleus into the mantle and visceral mass. Therefore, the study of the different response mechanisms of *H. cumingii* after the insertion surgery of mantle and visceral masses is of great theoretical and practical significance for production.

Bone morphogenetic proteins (BMPs) belong to the transforming growth factor-beta (TGF-β) superfamily and are synthesized as secreted signaling factors with additional prepeptide sequences (8). BMPs have attracted much attention in higher organisms, and are associated with bone formation and differentiation, regulation of reproduction, cell proliferation, migration and differentiation (9–11). BMPs are osteogenic inducers that play an important role in bone formation, limb formation, osteoblast differentiation and embryonic kidney development. Jiang et al. (12) observed the effects on zebrafish bone metabolism after inhibition of *BMP2a* expression by knockdown. The conclusion showed that zebrafish *BMP2* was down-regulated by knockdown technique, it was visualized that zebrafish had delayed bone development, and *BMP2* played a crucial role in zebrafish bone metabolism. By cloning the *BMP4* gene of *Siniperca chuatsi* and analyzing its expression, Cao et al. (13) demonstrated that *BMP4* is involved in the regulation of jaw remodeling, and the development of jaw remodeling is correlated with its feeding behaviors at different stages. In higher organisms, *BMP11* can inhibit the progression of atherosclerosis by activating TGF-β/Smad2/3, AMPK/eNOS signaling pathways and inhibiting JNK and NF-κB signaling pathways (14). In addition, BMPs are involved in the biomineralization process of mollusks. *BMP3* is involved in the shell formation of *Pinctada fucata*, as well as calcium ion metabolism in the gills (15). *BMP10* is involved in the formation of shells and pearls of *H. cumingii*, which plays an important role in the calcium carbonate-mediated biomineralization reactions and may be involved in calcium ion uptake and metabolism (16).

In the present study, we identified the BMP gene family members in *H. cumingii* from the genome database and used bioinformatics methods to study their phylogenetic relationships, motif composition, gene structure and chromosomal localization. Moreover, qRT-PCR was used to examine the relative expression of BMP genes before and after the insertion to the mantle and visceral masses. This study provides a theoretical basis for further studies on the different roles played by the BMP gene family in the mantle and visceral mass of *H. cumingii* after insertion.

## 2 Materials and methods

### 2.1 Experimental animals and sample collection

Experimental animals: One hundred healthy 2-year-old *H. cumingii* of uniform body size (length: 12 ± 2 cm, width: 7 ± 1 cm, height: 2.5 ± 0.8 cm, weight: 225.23 ± 30.77 g) purchased from the aquaculture farm in Jinhua City, Zhejiang Province, China were randomly selected and temporarily cultivated in the laboratory. Twenty were assigned to the control group without insertion and 80 were assigned to the experimental group, small pieces of donor mantle (2 mm × 2 mm) and 3 mm diameter nucleus were inserted in the posterior part of the host mantle and visceral mass (17). After the insertion surgery, the mussels were temporarily cultivated in a laboratory tank with 100 L circulating water, and the water temperature was maintained at about 26°C. Chlorella was fed twice a day.

Sample collection: Six mussels were collected at five time points (0, 5, 20, 50, and 90 d), respectively. The mussels were killed by cutting off adductor muscle, 80–100 mg of samples were collected from the insertion sites of mantle and visceral mass immediately, and were frozen by liquid nitrogen and stored in a refrigerator at −80°C.

### 2.2 Genome-wide identification of BMP genes

The whole genome sequencing data of the *H. cumingii* (GCA_028554795.2) had been obtained by laboratory in the early stage (18). The Hidden Markov Model file of BMP genes (PF00019 and PF00688) was downloaded from the Pfam protein family database^1^ and the amino acid sequences of the genome of *H. cumingii* were searched by hmmsearch function of the TBtools software. Subsequently, structural domain validation was performed using NCBI-CDD^2^ and SMART^3^. The BMP gene family of *H. cumingii* was finally confirmed and named based on NCBI-BLAST^4^ results, combined with genome-wide annotation. The number of amino acids (aa), molecular weight (MW), isoelectric point (pI), stability index and grand average of hydrophilicity (GRAVY) of BMP family proteins were predicted by ExPASy^5^. Subcellular localization of BMP gene family members was predicted using the Euk-mPLoc2.0^6^. The secondary structures of BMP family proteins were analyzed using the PSORT II^7^.

### 2.3 Phylogenetic analysis of BMP gene family

Amino acid sequences of typical species (*Homo sapiens*, *Danio rerio*, *Crassostrea gigas*, *Crassostrea virginica*, and *Mizuhopecten yessoensis*) were obtained from the NCBI database^8^ and saved in fasta format. All protein accession numbers and sequences used for the analysis were shown in Supplementary Table S1. The amino acid sequences were imported into MEGA 11 software and the multiple sequence alignment of full-length proteins among species was assessed using MUSCLE. An phylogenetic tree was constructed using the Neighbor-Joining (NJ) method and the bootstrap replications were 1,000. The phylogenetic tree was visually improved using iTOL^9^.

### 2.4 Gene structure analysis

The conserved domains and motifs of the BMP gene family members were predicted using the NCBI-CDD and MEME^10^, respectively. The maximum number of motifs was set 10. The BMP protein sequences of the *H. cumingii* were subjected to multiple sequence comparison analysis using the MEGA 11 software (19), and the phylogenetic tree was constructed by the Maximum Likelihood (ML) method (bootstrap replications of 1,000). The visualization of the gene structure of BMP family members were applied by TBtools software (v 2.096) (20) based on the genome annotation file.

### 2.5 Chromosomal location analysis

BMP genes were mapped to chromosomes based on the chromosomal position provided in the *H. cumingii* genome database. Chromosomal location of 12 BMP genes was visualized by TBtools software (v 2.096).

### 2.6 Quantitative real-time PCR (qRT-PCR) of BMP gene family

Total RNA was extracted from the mantle and visceral mass of each mussel at five time points by using the Trizol Reagent (TaKaRa, Tokyo, Japan). The quality of RNA was tested by 1% agarose gel electrophoresis, and the concentration of total RNA and A260/A280 ratio were examined on a NanoDrop 2000 (Thermo, Waltham, MA, USA). cDNA was synthesized using a *Evo M-MLV* Reverse Transcription Mix Kit (Accurate Biology, Hunan, China) according to the manufacturer’s protocol. Specific primers for qRT-PCR were designed using Premier Primer 5.0 (Table 1). Follow the instructions of TB Green® Premix Ex Taq™ (TaKaRa, Tokyo, Japan) for qRT-PCR. The amplification was performed in triplicate on a Bio-Rad CFX96 (Bio-Rad, Hercules, CA, USA), which was consistent with the method reported by Shen et al. (21). Relative gene expression data was analyzed using the 2^−ΔΔCt^ method to obtain the expression of each sample relative to the internal reference gene EF1α. All reactions were performed in three replicates using six biological samples. t-test and one-way ANOVA were performed using SPSS 27.0. The means among various groups were compared by Duncan’s multiple range tests. The results are presented as the means ± SEM, and the statistical significance is represented by *p* < 0.05.

**Table 1:** The primers sequences of qRT-PCR.

Primer name	Sequence (5′–3′)
*EF1α*-F	GGAACTTCCCAGGCAGACTGTGC
*EF1α*-R	TCAAAACGGGCCGCAGAGAAT
*BMP2a*-F	GCCAGAGATGACCACAGAA
*BMP2a*-R	ATTCGCCAGCACAATAGTATG
*BMP2b*-F	CAGGCAAGAAGACAACAGAT
*BMP2b-*R	TGGATTAGTAGCACTCATAAGG
*BMP3*-F	GTGGAAGGTTATGTAGCAAGAA
*BMP3*-R	CTGGATTGTGGCGTGATTAG
*BMP5a*-F	GCCAGCAAGGAGACAAGA
B*MP5a-*R	TGAACGACACCAGGAAGAC
*BMP5b*-F	GAGATGAGAACTGCGTATGC
*BMP5b-*R	TGCTTCCGTCTTGCTTGT
*BMP7a*-F	CCAGAACTCTTCACCAACAA
*BMP7a-*R	AACACTCGCCTCCACAAT
*BMP7b*-F	GACAGAAGAAGAAGACACAGAG
*BMP7b*-R	GAACACTCGCCTCCACAA
*BMP7c*-F	GACGACGAAGAAGAAGAAGATA
*BMP7c*-R	GCTGTTGTTCACTGCTGTT
*BMP9-*F	AGGCAGCAGGTCAGACAT
*BMP9*-R	CTCCACAGACATACGCATTG
*BMP10a*-F	CAGCAGGTCAGAGATGTCA
*BMP10a*-R	AATATCCGAACCAAGCAGTG
*BMP10b*-F	TTGGCACACCTGGATTATTG
*BMP10b-*R	CCACTACCATTCCGTCGTA
*BMP11*-F	AGAAGAATACTACGACGAAGAC
*BMP11*-R	GGAGGTAGCAACTTATAGACAA

## 3 Results

### 3.1 Identification of BMP genes in *H. cumingii*

As shown in Table 2, 12 BMP gene family members, named *BMP2a*, *BMP2b*, *BMP3*, *BMP5a*, *BMP5b*, *BMP7a*, *BMP7b*, *BMP7c*, *BMP9*, *BMP10a*, *BMP10b*, *BMP11* were identified within the genome of *H. cumingii* in this study. The length of ORF (open reading frames) ranged from 510 to 1,752 bp. The longest sequence was *Hc-BMP7c*, encoding 583 amino acids, and the shortest sequence was *Hc-BMP10a*, encoding 169 amino acids. The corresponding protein MW ranged from 19.32 to 65.99 kDa, and the pI ranged from 4.73 to 9.31. Four BMPs proteins (*BMP5a*, *BMP7b*, *BMP7c*, and *BMP11*) were acidic proteins (pI < 7.5), and the rest of BMPs proteins were alkaline proteins (pI > 7.5). All of the 12 BMPs proteins had an instability index greater than 40 and the GRAVY of proteins were all negative, indicating that the family proteins were unstable hydrophilic proteins.

**Table 2:** Characteristics of *BMP* genes in *H. cumingii.*

Gene name	Number of amino acid	Molecular weight (kDa)	Theoretical pI	Instability index	Aliphatic index	Grand average of hydropathicity
*Hc-BMP2a*	188	21,748.42	8.54	47.58	84.57	−0.181
*Hc-BMP2b*	368	40,945.01	8.64	39.47	79.78	−0.355
*Hc-BMP3*	485	55,614.31	9.31	53.36	71.79	−0.66
*Hc-BMP5a*	528	59,198.38	6.21	51.78	74.41	−0.49
*Hc-BMP5b*	306	34,163.56	8.86	42.63	80.95	−0.202
*Hc-BMP7a*	433	50,094.07	8.63	49.35	76.35	−0.575
*Hc-BMP7b*	420	48,326.78	6.33	49.15	76.57	−0.529
*Hc-BMP7c*	583	65,989.51	4.73	53.73	71.46	−0.81
*Hc-BMP9*	239	26,926.1	9.3	51.74	86.4	−0.295
*Hc-BMP10a*	169	19,324.43	9.28	49.35	86.45	−0.132
*Hc-BMP10b*	454	51,498.3	7.63	45.17	88.22	−0.431
*Hc-BMP11*	376	43,827.59	5.72	40.33	80.35	−0.645

The subcellular localization and secondary structures of BMP gene family proteins were analyzed, as shown in Table 3. *BMP2a* was distributed in the cytoplasm, *BMP7b* was distributed in the endoplasmic reticulum and *BMP10a* was distributed in the mitochondria, while the rest of BMPs were distributed in the nucleus. The results of secondary structure analysis showed that 12 BMPs proteins were dominated by random coil and α-helix structures, with a percentage ranging from 62.34 to 81.91%, while β-turn accounted for the lowest percentage (2.13–11.30%).

**Table 3:** The subcellular localization and secondary structure prediction of *BMP* proteins in *H. cumingii.*

Gene name	Subcellular localization	Alpha helix (%)	Extended strand (%)	Beta turn (%)	Random coil (%)
*Hc-BMP2a*	Cytoplasm	36.17	15.96	2.13	45.74
*Hc-BMP2b*	Nucleus	25.00	21.20	7.07	46.74
*Hc-BMP3*	Nucleus	28.87	16.70	2.06	52.37
*Hc-BMP5a*	Nucleus	34.85	17.23	8.33	39.58
*Hc-BMP5b*	Nucleus	25.49	18.95	5.56	50.00
*Hc-BMP7a*	Nucleus	26.56	18.94	3.46	51.04
*Hc-BMP7b*	Endoplasmic reticulum	19.76	21.67	2.38	56.19
*Hc-BMP7c*	Nucleus	28.47	18.70	5.32	47.51
*Hc-BMP9*	Nucleus	23.01	26.36	11.30	39.33
*Hc-BMP10a*	Mitochondria	34.32	20.12	6.51	39.05
*Hc-BMP10b*	Nucleus	23.35	18.28	3.74	54.63
*Hc-BMP11*	Nucleus	34.57	17.55	2.39	45.48

### 3.2 Phylogenetic analysis of the BMP genes

**Figure 1:**
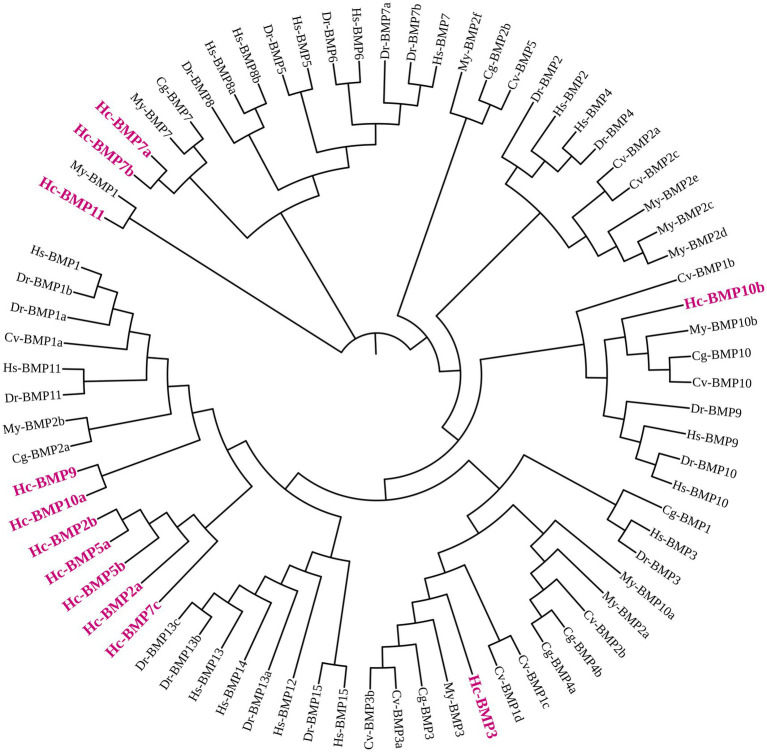
Phylogenetic tree of the BMP gene family in *H. cumingii* (Hc) and representative species. *H. sapiens* (Hs), *D. rerio* (Dr), *C. gigas* (Cg), *C. virginica* (Cv), and *M. yessoensis* (My). The phylogenetic tree was constructed using the Neighbor-Joining (NJ) method with 1,000 bootstrap replicates.

To investigate the phylogenetic relationships of BMP genes in *H. cumingii*, a phylogenetic tree was constructed (Figure 1) using BMP amino acid sequences from *H. sapiens*, *D. rerio*, *C. gigas*, *C. virginica*, and *M. yessoensis*. *BMP2a*, *BMP2b*, *BMP5a*, *BMP5b*, and *BMP7c* were clustered into one branch by themselves, *BMP9* and *BMP10a* were clustered into one branch, all of which were phylogenetically distant from other higher organisms and shellfish. *BMP3* and *BMP10b* were clustered into one branch with *C. gigas*, *C. virginica*, and *M. yessoensis*, which indicated that *BMP3* and *BMP10b* are phylogenetically closely related between *H. cumingii* and other shellfish. *BMP7a* and *BMP7b* were clustered with *C. gigas* and *C. virginica*, which were phylogenetically close to each other. *BMP11* was clustered with *BMP1* of *M. yessoensis* and its phylogenetic relationship needs to be further investigated.

### 3.3 Gene structures analysis of the BMP genes

**Figure 2:**
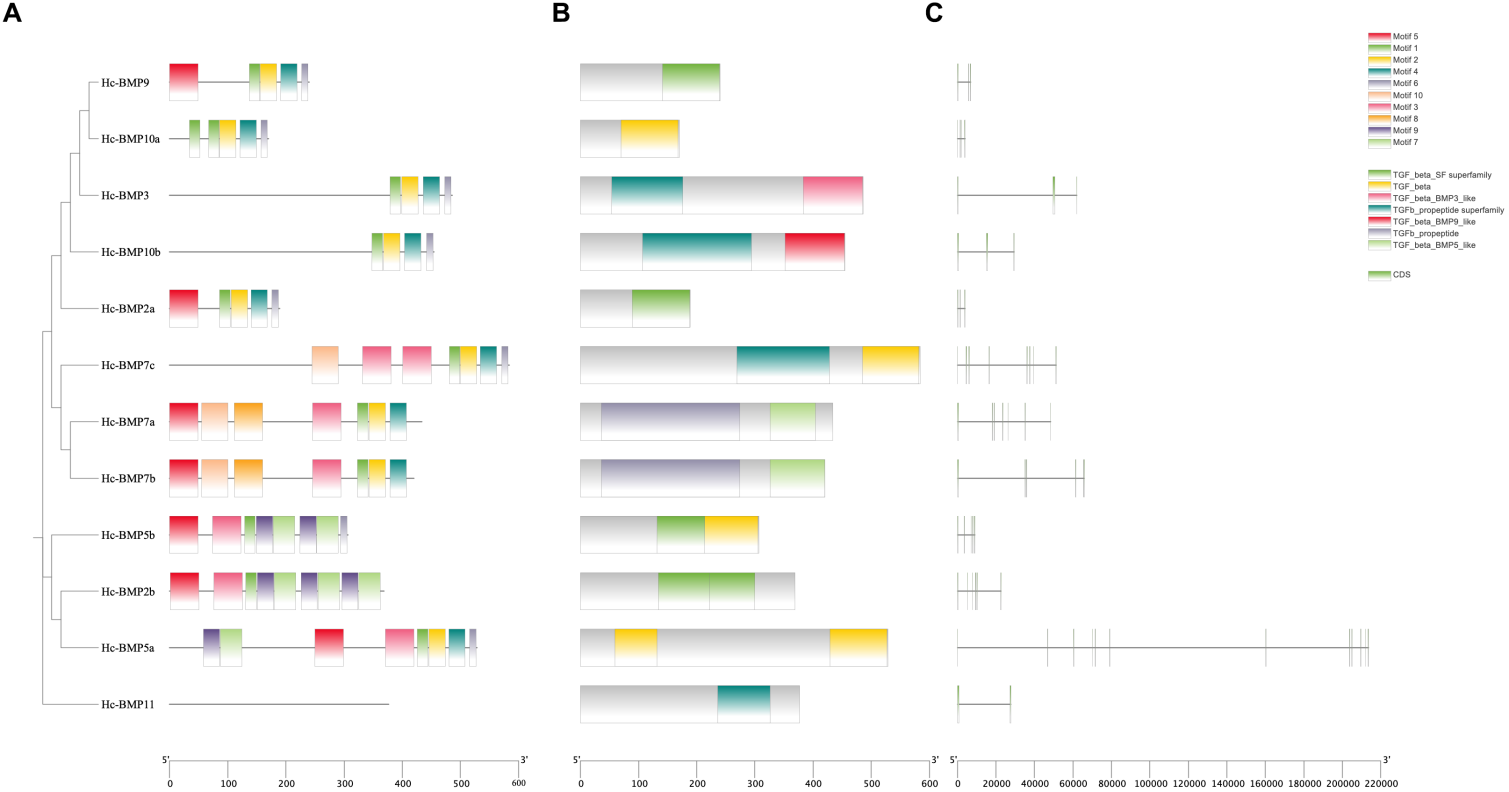
Phylogenetic tree, conserved motifs, conserved domains and gene structures of BMP genes in *H. cumingii*. **(A)** phylogenetic tree and conserved motifs, **(B)** conserved domains, and **(C)** gene structures.

Analysis of the conserved motifs of BMP proteins (Figure 2A; Table 4) showed that the amino acid sequence length of motifs ranged from 12 to 50 aa. Motif 1, Motif 2, and Motif 4 were highly conserved, which were shared by 11 BMP genes, followed by Motif 6, and fewer family members contained Motif 7, Motif 8, Motif 9, and Motif 10. The distribution of conserved motifs was similar among BMP genes with near positions in the phylogenetic tree.

**Table 4:** Conserved motifs information of *BMP* proteins in *H. cumingii.*

Motif	Protein sequence	AA number	Gene number
Motif 1	GQACQRHPLYVNFTSJGWN	19	11
Motif 2	WIIAPKGYAAFYCAGECNFPLGDLLNPKH	29	11
Motif 3	PGQETBPGKSGTVGDDGPENKQPFMTAMFKMSKEEYLRETLSARKRRHSS	50	6
Motif 4	JHSSEPPDIPKPCCVPTKLSSJSVLYYIN	29	11
Motif 5	MHAVGSLTWRILLSLTCIIHLYKEFSCESIVHKTDNGLEQNIPENQMPGM	50	7
Motif 6	YQNMVVEECGCR	12	8
Motif 7	AKIRGLLSATNPBIPKLCCVPTKLSAINAJTNINSTVE	38	3
Motif 8	FNNNPVPDINEINGTDJIMSFVNHARKLPFLRHERDRMFYFDFSDVSPGE	50	2
Motif 9	WLVAPPGYMSYYCAGECNIVSADLIRTKH	29	3
Motif 10	QQEILALLGLHHRPNPVKNNAVEKSAPQFMLELYNKIRSDDDDETP	46	3

Analysis of the conserved domains of BMP genes (Figure 2B) showed that most of BMP genes possessed the TGF-β SF superfamily and TGF-β superfamily. The distribution of conserved domains was similar among BMP genes with near positions in the phylogenetic tree. As shown in the figure, Motif 1, Motif 2, Motif 3, and Motif 4 are involved in the formation of the TGF-β SF superfamily, and Motif 1, Motif 2, Motif 4, and Motif 6 are involved in the formation of the TGF-β superfamily. These conserved motifs may perform the functions of the corresponding structural domains.

Analysis of the gene structure of BMP genes (Figure 2C) showed that the total length of the different genes varied, with *BMP2a* being the shortest and *BMP5a* being the longest. In addition, similarity of gene structure among BMP proteins with near positions in the phylogenetic tree can be seen from the figure.

### 3.4 Chromosomal localization

**Figure 3:**
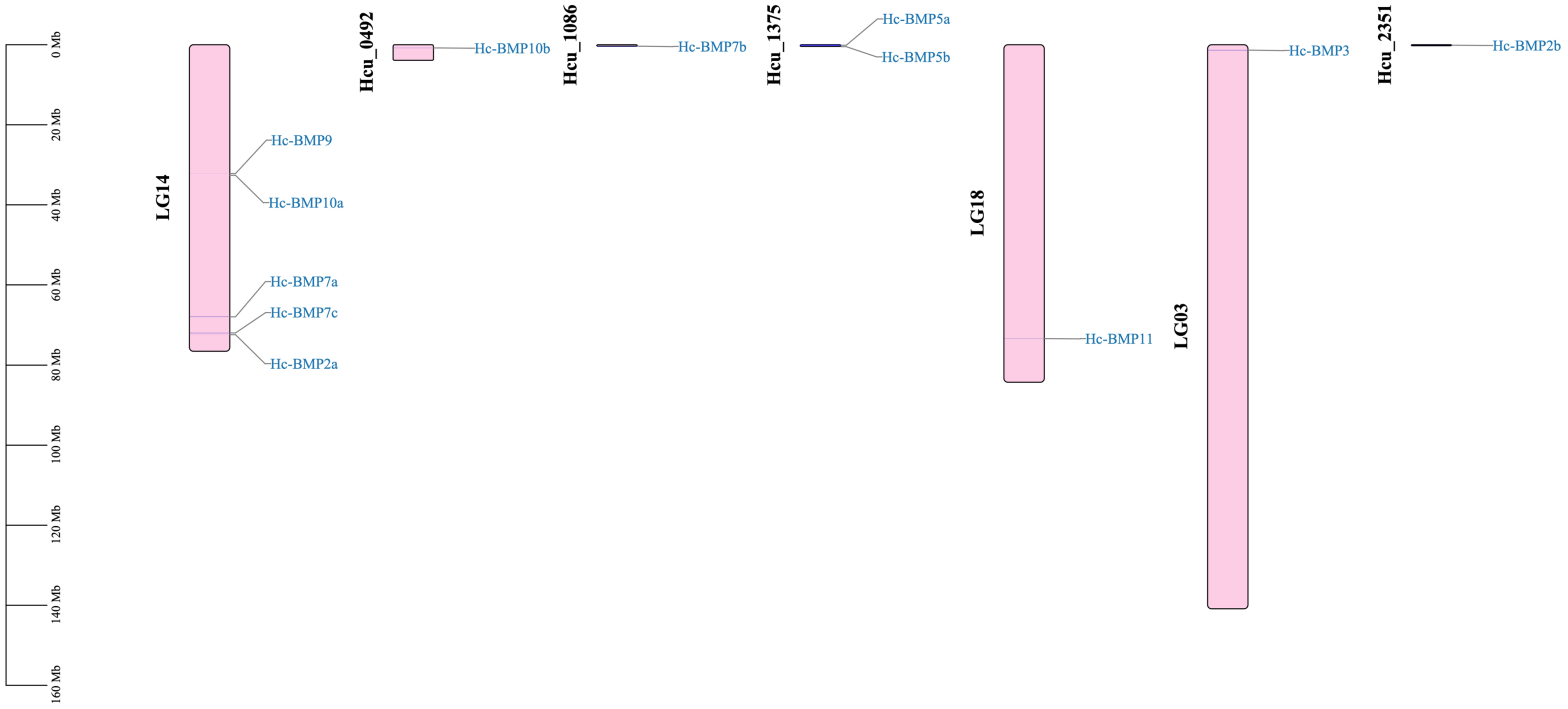
Distribution of the BMP genes on chromosomes.

The localization of the BMPs gene family on the chromosomes is shown in Figure 3. The number of chromosomes in the *H. cumingii* is 2n = 38. Seven genes (*BMP2a*, *BMP3*, *BMP7a*, *BMP7c*, *BMP9*, *BMP10a*, and *BMP11*) were located to four different chromosomes, and five genes (*BMP2b*, *BMP5a*, *BMP5b*, *BMP7b*, and *BMP10b*) were not localized on chromosomes. Chromosome 14 contained five BMP genes, and chromosomes 18 and 3 contained one BMP gene, respectively. Meanwhile, *BMP9* and *BMP10a* were located in the middle part of chromosome 14, while the other family members were distributed on both ends of the chromosomes. The scale is in megabases (Mb).

### 3.5 Expression analysis

**Figure 4:**
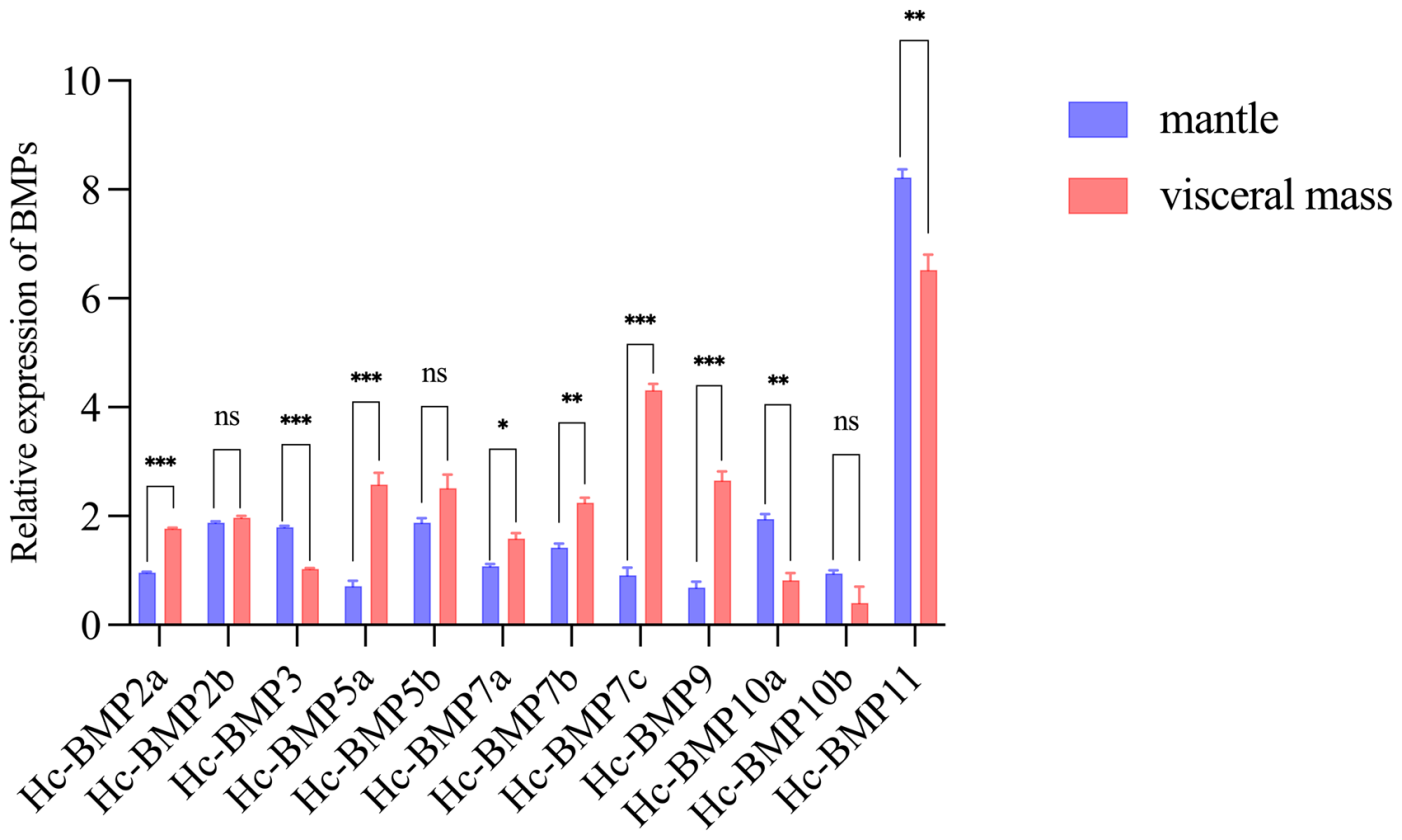
Relative expression of HcBMPs in the mantle and visceral mass without insertion. *p < 0.05, **p < 0.01, ***p < 0.001, ns means not significant.

qRT-PCR was performed to detect the expression of 12 BMP genes in the mantle and visceral mass of *H. cumingii*, which were not inserted. As shown in Figure 4, except for *BMP2b*, *BMP5b*, and *BMP10b*, the expression of the other BMPs genes in the mantle and visceral mass were significantly different (*p* < 0.05), this research found significant tissue specificity in BMPs gene expression. Among them, the expression of *BMP2a*, *BMP5a*, *BMP7c*, and *BMP9* were significantly higher in the visceral mass than in the mantle (*p* < 0.001), and their expression in the visceral mass were 1.84 times, 3.68 times, 3.86 times and 4.74 times higher than in the mantle, respectively. In contrast, *BMP3*, *BMP10a* and *BMP11* expression in the mantle were significantly higher than in the visceral mass, especially the expression of *BMP10a* in the mantle was 2.37 times higher than in the visceral mass.

**Figure 5:**
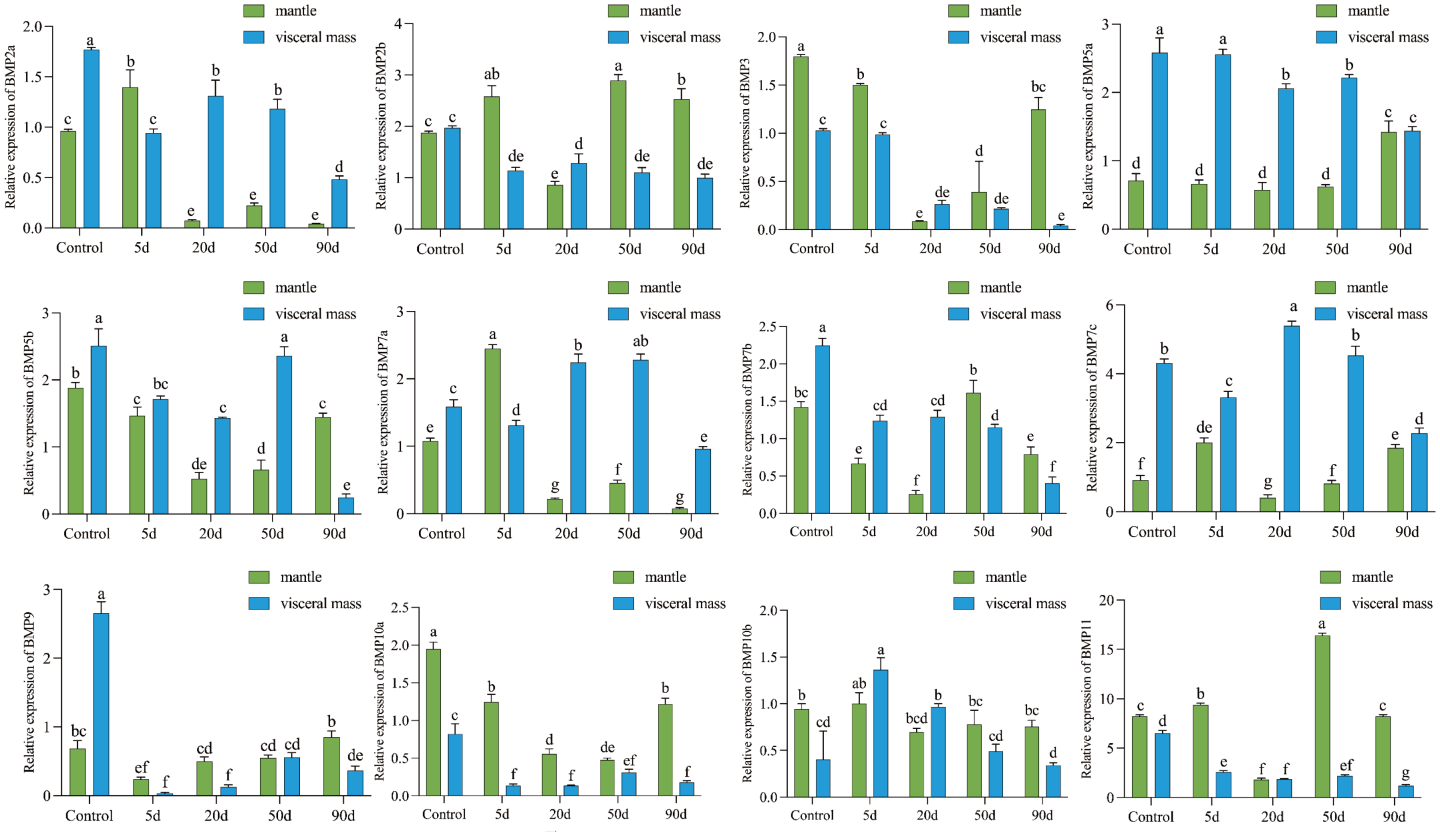
Relative expression of HcBMPs in the mantle and visceral mass after insertion. Different letters indicate significant differences between groups (*p* < 0.05).

qRT-PCR was performed to detect the expression changes of 12 BMP genes at 5, 20, 50, and 90 d after the insertion surgery. As shown in Figure 5, the expression of all 12 BMP family members was significantly different (*p* < 0.05) after the mantle and visceral mass were inserted. Compared with the control group, BMP genes showed different expression patterns during the insertion of nucleus for pearl cultivating, except for *BMP7c*, *BMP10a*, and *BMP11*. Among them, the expression of *BMP2a* was significantly higher in mantle than in visceral mass at 5 d, unlike the control and other time points, and the expression trends of *BMP7a* and *BMP2a* were basically the same. The expression of *BMP3* was higher in the visceral mass at 20 d, and the expression pattern of *BMP10b* was opposite to that of the control at 5 d and 20 d, with the expression in visceral mass significantly higher than that of mantle. In the early stage, the of *BMP5a* and *BMP5b* were both significantly higher in visceral mass than in mantle, while at 90 d *BMP5a* showed no significant difference between the two, and *BMP5b* showed significantly higher expression in mantle than in visceral mass. *BMP7b* had significantly higher expression in mantle than in visceral mass at 50 d and 90 d, unlike the early stage. In addition, the expression pattern of *BMP2b* and *BMP9* changed greatly after insertion, *BMP2b* showed no significant difference between the expression of mantle and visceral mass in the control group, while the expression in mantle was higher at all time points after insertion except for the higher expression in visceral mass at 20 d, and *BMP9* expression was significantly higher in mantle than in visceral mass at all time points after insertion, in contrast to the control group, except at 50 d when there was no significant difference between the two.

## 4 Discussion

BMPs are key factors in the biomineralization of pearl mussels, but their roles in pearl culture are less reported. Because of the functional diversity of BMP gene family, in-depth studies have been conducted in several species, with 44 BMP genes identified from *Cyprinus carpio* (22), 11 BMP genes from Chinese soft-shell turtle (*Pelodiscus sinensis*) (23), 26 BMP genes were identified from *Cynoglossus semilaevis* (24), and 18 BMP genes from *Hypophthalmichthys nobilis* (25). In this study, 12 BMP gene family members were identified from the genome of *H. cumingii*, including two *BMP2* members, one *BMP3* member, two *BMP5* members, three *BMP7* members, one *BMP9* member, two *BMP10* members and one *BMP11* member. The BMP gene family identified in this study is complete, which lays the foundation for subsequent functional and evolutionary analyses. Analysis of the physicochemical properties of the BMP gene family members showed that the ORF lengths were shorter and the corresponding protein molecular weights were smaller compared to those of other species (26). Moreover, the pI of most BMP proteins was greater than 7.5, proving that they may be a class of basic proteins. Subcellular localization revealed that most of the BMPs were present in the nucleus, but also in the mitochondria, cytoplasm, and endoplasmic reticulum, which is inconsistent with the findings of BMPs from *Rachycentron canadum* (27), where most of the BMPs exist outside the cell. Structural analysis demonstrated that their secondary structures contain a higher number of α-helices and random coil, suggesting that α-helix and random coil have a greater influence on the function of BMP family proteins of *H. cumingii*.

By constructing a phylogenetic tree of *H. cumingii* with higher organisms (human, zebrafish) and other shellfish (Pacific oyster, American oyster, and scallop), it was found that the *H. cumingii* BMPs sequences were distantly phylogenetically related to the higher organisms and none of them clustered into a single branch, whereas some of the BMP genes clustered into a single branch with other shellfish, and some of them clustered into a single branch on their own. Many literatures (28–30) suggest that the evolutionary relationship of BMP genes in molluscs is closer to that of other molluscs and more distantly related to higher organisms (human and zebrafish), which is consistent with the results of present study. However, the phylogenetic analyses of BMP gene family in other species (*H. nobilis*, *R. canadum*, *C. carpio*, and *T. dalaica*) all classified the BMP gene family into five subfamilies (22, 25, 31, 32): *BMP2/4/16*, *BMP5/6/7/8*, *BMP9/10*, *BMP12/13/14*, and *BMP1/3/11/15*, but the phylogenetic analysis carried out in the present study could not come to this conclusion, suggesting that the phylogenetic pattern of BMP gene family in *H. cumingii* is worthy of further in-depth study. Statistical analysis of the conserved motifs in the BMP gene family revealed that Motif 1, Motif 2, and Motif 4 were the most conserved, 11 genes contained these motifs, suggesting that they may be the key factors in the functioning of structural domains of BMPs. Members of different branches have different motif compositions, and these conserved motifs may be the main reason why different BMP genes are involved in different biological functions. Structural domain analysis showed that most of BMP gene family members in this study have the TGF-β SF superfamily and TGF-β superfamily structural domain, which has been proven to be related to the formation and repair of shells. Both *BMP3* and *BMP10* of *Pinctada martensi* have the TGF-β structural domain, which is associated with damage repair of shells (15), *BMP2* and *BMP10* of *H. cumingii* have also been demonstrated to be related to the formation of the prismatic layer and pearl layer of shells as well as calcium metabolism (16). TGF-β of *H. cumingii* also possesses this structural domain, which was found to be related to immune response and wound repair (33). The above results all suggest that the BMP gene family may play an important role in the biomineralization process of pearl mussels.

Many studies have shown that nucleus insertion to *H. cumingii* causes changes in the expression of relevant biomineralization genes in response to wound repair (34–36). Since most of the BMP genes showed significant differences of expression between mantle and visceral mass, it is hypothesized that these genes perform different functions in the mantle and visceral mass. We suggested that BMPs have different mechanisms of action after insertion surgery of the mantle and visceral mass, so the insertion surgery was performed. We focused on the differences of BMP expression in mantle and visceral mass after nucleus insertion for pearl cultivating. Except for *BMP7c*, *BMP10a*, and *BMP11*, the expression pattern of other BMP genes in mantle and visceral mass after insertion were changed. The trend of *BMP2a* and *BMP7a* expression change before and after insertion in mantle and visceral mass were basically the same, the expression of both genes in mantle significantly increased at 5 d, which indicated that these two genes played a role 5 days after mantle insertion. But *BMP2a* and *BMP7a* expression decreased significantly in the late stage of mantle insertion, which may be suppressed during the pearl formation process. Interestingly, the phylogenetic tree showed that their evolutionary relationship was not close, which might be due to the fact that their action mechanisms during pearl cultivating were similar, whereas the expression of *BMP7b* increased significantly at the late stage of mantle insertion. *BMP7a* and *BMP7b* may perform different functions after mantle and visceral mass insertion. Meanwhile, Lin (37) demonstrated that *BMP7* was involved in the formation of nacreous layer and prismatic layer of *H. cumingii*, and Fan et al. (38) found that *BMP7* may play an important role in the shell formation and repair process of *P. martensi*, suggesting that *BMP7* can indeed participate in the biomineralization of mantle of the pearl mussel. We suppose that *BMP2* and *BMP7* play an important role in the biomineralization of mantle and that the difference in the roles of two homologous genes, *BMP7a* and *BMP7b*, is of interest. There are literatures (15) suggesting that the short-term expression of *BMP3* in *P. martensi* increased significantly during damage repair of shells, which was presumed to be involved in the damage repair of shells. Duan (16) showed that the expression of *BMP10* in *H. cumingii* mantle increased significantly after 7 days of mantle insertion. Both indicated that *BMP3* and *BMP10* play important roles in the biomineralization of mantle. In our study, we found that *BMP3* and *BMP10a* were expressed at higher levels and with similar expression trends after mantle insertion, suggesting that they play an important role in pearl cultivating by mantle insertion. However, the specific mechanism still needs to be further investigated. Both *BMP5a* and *BMP5b* were expressed at higher levels after visceral mass insertion, with *BMP5b* expression being suppressed after 90 days of visceral mass insertion. This finding implies that *BMP5a* and *BMP5b* play a role in pearl cultivating by visceral insertion. But there is less literature available on mollusc *BMP5*, and only in higher organisms has it been shown to be related to bone formation and trauma repair (39, 40). Therefore, the function of *BMP5* in the pearl cultivating of *H. cumingii* requires further study. *BMP9* expression was significantly decreased 5 days after nucleus insertion in the visceral mass, and it may be suppressed in the short-term inflammatory and biomineralization. *BMP9* was also not found to be studied in mollusks, but higher organisms’ studies have shown that its expression induces osteoblast differentiation, which is found to be related to bone formation and repair (41, 42). *BMP9* may perform opposite functions in higher organisms and shellfish, and the specific mechanisms need to be further investigated. The differential expression patterns of BMP in mantle and visceral mass of the mussel after insertion indicate that the BMP gene family plays different roles in the pearl-cultivating process of mantle and visceral mass, some of which are promoted and some of which are suppressed. A more in-depth study of the above genes can provide a theoretical basis for shortening the pearl-culture cycle of *H. cumingii* and improving the quality of pearls.

## 5 Conclusion

In this study, bioinformatics analysis of BMP genes was performed based on genome of *H. cumingii*. A total of 12 BMP genes were identified, which were distributed on four chromosomes. The phylogenetic tree showed that HcBMPs are evolutionarily close to other shellfish, but evolutionarily distant from higher organisms (human and zebrafish). qRT-PCR results showed that the HcBMPs were differentially expressed in the mantle and visceral mass, and the expression level was higher in the visceral mass. This research found significant tissue specificity in BMPs gene expression. Further insertion surgery in the mantle and visceral mass significantly alters the expression profiling of the BMP gene family. *BMP2a*, *BMP5a*, *BMP5b*, *BMP7a*, and *BMP7c* had higher expression after visceral mass insertion, whereas *BMP2b*, *BMP3*, *BMP9*, and *BMP10a* had higher expression after mantle insertion, suggesting that HcBMPs may play different roles during the insertion of nucleus in the mantle and visceral mass, respectively. The present study lays the foundation and data support for the preliminary elucidation of regulatory role and mechanism of HcBMPs in the pearl-cultivating process of mantle and visceral mass.

## Data availability statement

The original contributions presented in the study are included in the article/Supplementary material, further inquiries can be directed to the corresponding authors.

## Ethics statement

The animal study was reviewed and approved by Shanghai Ocean university. The study was conducted in accordance with the local legislation and institutional requirements.

## Author contributions

YC: Methodology, Writing – original draft, Writing – review & editing. SL: Conceptualization, Writing – review & editing. YY: Investigation, Methodology, Writing – original draft. JS: Investigation, Writing – original draft. XC: Writing – original draft, Software. XY: Visualization, Writing – original draft. XX: Supervision, Writing – review & editing. XB: Conceptualization, Project administration, Supervision, Writing – review & editing, Funding acquisition, Resources. WL: Conceptualization, Project administration, Supervision, Writing – review & editing, Resources.

## Footnotes

^1^http://pfam.xfam.org/

^2^https://www.ncbi.nlm.nih.gov/Structure/bwrpsb/bwrpsb.cgi

^3^http://smart.embl-heidelberg.de

^4^https://blast.ncbi.nlm.nih.gov/Blast.cgi

^5^http://web.expasy.org/compute_pi/

^6^http://www.csbio.sjtu.edu.cn/bioinf/euk-multi-2/

^7^https://psort.hgc.jp/form2.html

^8^https://www.ncbi.nlm.nih.gov

^9^https://itol.embl.de/

^10^https://meme-suite.org/meme/tools/meme
