# Supplementary material for: Identification and expression profiling of the bone morphogenetic protein gene family based on pearl culture in mantle and visceral mass of Hyriopsis cumingii
Source: Front Vet Sci. 2024 Aug 21;11:1445594. doi: 10.3389/fvets.2024.1445594 (PMC11373570; doi:10.3389/fvets.2024.1445594)
Supplement: Supplementary file 1 [file Table_1.DOCX]

**Table S1. The BMP gene information for phylogenetic analysis**

| **Number** | **Species** | **Protein Name** | **Accession** |
| --- | --- | --- | --- |
| **1** | Homo sapiens | BMP1 | NP_001190.1 |
| **2** | Homo sapiens | BMP2 | NP_001191.1 |
| **3** | Homo sapiens | BMP3 | NP_001192.4 |
| **4** | Homo sapiens | BMP4 | NP_001193.2 |
| **5** | Homo sapiens | BMP5 | NP_066551.1 |
| **6** | Homo sapiens | BMP6 | NP_001709.1 |
| **7** | Homo sapiens | BMP7 | NP_001710.1 |
| **8** | Homo sapiens | BMP8a | NP_861525.2 |
| **9** | Homo sapiens | BMP8b | NP_001711.2 |
| **10** | Homo sapiens | BMP9 | NP_057288.1 |
| **11** | Homo sapiens | BMP10 | NP_055297.1 |
| **12** | Homo sapiens | BMP11 | NP_005802.1 |
| **13** | Homo sapiens | BMP12 | NP_878248.2 |
| **14** | Homo sapiens | BMP13 | NP_001001557.1 |
| **15** | Homo sapiens | BMP14 | NP_000548.2 |
| **16** | Homo sapiens | BMP15 | NP_005439.2 |
| **17** | Danio rerio | BMP1a | NP_001035126.1 |
| **18** | Danio rerio | BMP1b | NP_001034901.1 |
| **19** | Danio rerio | BMP2 | NP_571434.1 |
| **20** | Danio rerio | BMP3 | NP_001071233.1 |
| **21** | Danio rerio | BMP4 | NP_571417.1 |
| **22** | Danio rerio | BMP5 | NP_957345.1 |
| **23** | Danio rerio | BMP6 | NP_001013357.1 |
| **24** | Danio rerio | BMP7a | NP_571396.1 |
| **25** | Danio rerio | BMP7b | NP_001070614.2 |
| **26** | Danio rerio | BMP8 | NP_001038436.1 |
| **27** | Danio rerio | BMP9 | NP_001165057.2 |
| **28** | Danio rerio | BMP10 | NP_001124072.1 |
| **29** | Danio rerio | BMP11 | NP_998140.1 |
| **30** | Danio rerio | BMP13a | XP_694563.2 |
| **31** | Danio rerio | BMP13b | NP_001153466.1 |
| **32** | Danio rerio | BMP13c | NP_571062.1 |
| **33** | Danio rerio | BMP15 | NP_001018320.1 |
| **34** | Crassostrea gigas | BMP7 | XP_011428157.1 |
| **35** | Crassostrea gigas | BMP3 | XP_011415620.2 |
| **36** | Crassostrea gigas | BMP4a | XP_011416937.2 |
| **37** | Crassostrea gigas | BMP4b | XP_011416938.2 |
| **38** | Crassostrea gigas | BMP1 | XP_011422522.2 |
| **39** | Crassostrea gigas | BMP2a | XP_011426448.2 |
| **40** | Crassostrea gigas | BMP2b | XP_011431554.2 |
| **41** | Crassostrea gigas | BMP10 | XP_034313024.1 |
| **42** | Crassostrea virginica | BMP1a | XP_022293378.1 |
| **43** | Crassostrea virginica | BMP1b | XP_022294141.1 |
| **44** | Crassostrea virginica | BMP1c | XP_022294684.1 |
| **45** | Crassostrea virginica | BMP1d | XP_022294683.1 |
| **46** | Crassostrea virginica | BMP2a | XP_022333696.1 |
| **47** | Crassostrea virginica | BMP2b | XP_022303871.1 |
| **48** | Crassostrea virginica | BMP2c | XP_022333695.1 |
| **49** | Crassostrea virginica | BMP5 | XP_022319034.1 |
| **50** | Crassostrea virginica | BMP3a | XP_022317707.1 |
| **51** | Crassostrea virginica | BMP3b | XP_022317161.1 |
| **52** | Crassostrea virginica | BMP10 | XP_022313556.1 |
| **53** | Mizuhopecten yessoensis | BMP1 | XP_021344813.1 |
| **54** | Mizuhopecten yessoensis | BMP2a | XP_021378874.1 |
| **55** | Mizuhopecten yessoensis | BMP2b | XP_021378134.1 |
| **56** | Mizuhopecten yessoensis | BMP2c | XP_021367002.1 |
| **57** | Mizuhopecten yessoensis | BMP2d | XP_021367001.1 |
| **58** | Mizuhopecten yessoensis | BMP2e | XP_021367000.1 |
| **59** | Mizuhopecten yessoensis | BMP2f | XP_021359200.1 |
| **60** | Mizuhopecten yessoensis | BMP3 | XP_021343121.1 |
| **61** | Mizuhopecten yessoensis | BMP7 | XP_021377502.1 |
| **62** | Mizuhopecten yessoensis | BMP10a | XP_021367579.1 |
| **63** | Mizuhopecten yessoensis | BMP10b | XP_021353852.1 |
